# Monthly Summary

**Published:** 1882-08

**Authors:** 


### MONTHLY SUMMARY.

*Hemorrhage and Gangrene from a Carious Tooth.*—In the St. Louis *Medical and Surgical Journal*, Dr. William A Byrd reports the following case: He was called to see a boy between seven and eight years of age who was suffering from gangrene of the left side of the face and neck, accompanied with severe hemorrhage. The child was very anæmic, with the left side of the face swollen, with an opening that would admit the point of a finger, opposite the submaxillary gland, another in the meatus auditorious, and another in the mouth opposite to the outside of the first molar tooth; from all these openings was flowing a dark, very offensive fluid, mixed with arterial blood, and also shreds of gangrenous connective tissue protruded from them. For some weeks the child had suffered from an offensive discharge from the left ear; and about two weeks before I saw him the first left molar tooth commenced aching and the jaw to swell. Subsequently a black spot appeared opposite the tooth, and at this spot the skin gave way giving vent to a dark, very offensive discharge. The swelling from the ear and the tooth coalesced and the absecss broke into the ear and mouth, causing a profuse discharge from each. In a day ot two blood was freely discharged from all three openings. The patient was etheaized, and it was discovered that the bleeding came from so many points that it would be impracticable to secure all of them without too great a loss of blood and shock to the patient. It was, therefore, decided to ligate the common carotid, which, was done with catgut. Through the incission a great quantity of gangrenous matter was removed as high as above the zygoma and down nearly to the clavicle. The cavity was carefully washed out with a solution of boracic acid and packed with oakum soaked in eucalyptol and vaseline. He ralied well and seemed brighter, but sank again, and died on the second day. Dr. Byrd thinks that the carious tooth was the origin of the trouble, and that if it had been attended to in time ths result would most likely have been different.

---

*The Dental Engine Modified for Surgical Use.*—Dr. Garretson, of Philadelphia, in an illustrated article (*Annals af Anatomy and Surgery*, March, 1882), describes a new mechanical adjuvant for surgical operations, which seems calculated to give great assistance in a certain class of operations, where the parts are difficult of access or a special nicety is required that is hardly to be obtained with the ordinary instruments of the surgeon's armamentarium. This appliance is in reality nothing but a modified dental engine, and those who have seen the latter in use can readily understand the mechanism of the former. For the wire cable of the dental engine is substituted a stell arm. An assistant turns the crank of the surgical engine, while in the dental the foot upon a treadle gives the propelling power. The engine, of which the best is Bonwill's, of Philadelphia, is supplied with instruments of various forms, such as circular saws, rose drills, bits, etc. The rapidity with which it accomplishes the object may be appreciated from the fact that an instrument can be made to make fifteen thousand revolutions in the minute. Dr. G. finds the engine is especially suited for removing neecosed bone, exostoses, carious portions of shafts, segments of the coccyx, in exposing the zygomatic and sphenomaxillary fossæ, and for trephining.—*Med. Record.*

